# 锆基金属有机骨架复合材料用于海水中短裸甲藻毒素的固相萃取

**DOI:** 10.3724/SP.J.1123.2024.02026

**Published:** 2024-09-08

**Authors:** Zongbao CHEN, Shiye XIE, Yongjun LIU, Wenmin ZHANG, Min FANG, Lan ZHANG

**Affiliations:** 1.闽江师范高等专科学校,福建福州 350108; 1. Minjiang Teachers College, Fuzhou 350108, China; 2.福州大学,福建福州 350116; 2. Fuzhou University, Fuzhou 350116, China

**Keywords:** 金属有机骨架, 短裸甲藻毒素, 固相萃取, 高效液相色谱-串联质谱, 赤潮, metal-organic frameworks (MOFs), brevetoxin-A, solid phase extraction (SPE), high performance liquid chromatography-tandem mass spectrometry (HPLC-MS/MS), red tide

## Abstract

短裸甲藻毒素(BTX-A)是由有害藻类短裸甲藻产生的次生代谢物,检测海水中的藻毒素可以预测赤潮的发生和增长。将BTX-A含量监测作为赤潮预警的因子之一可以有效提高赤潮预警的准确性,开发一种高效富集海水中BTX-A的样品前处理方法迫在眉睫。研究通过溶剂热法制备了具有较大比表面积(310.9 m^2^/g)、对BTX-A有较强吸附作用的微米级Zr-MOFs复合材料(SiO_2_@UiO-66),并将其作为固相萃取(SPE)填料,结合高效液相色谱-串联质谱(HPLC-MS/MS)技术,建立了一种检出限低(3.0 pg/mL)、线性范围宽(10.0~200.0 pg/mL)、重复性良好(RSD≤8.5%, *n*=6)的海水中BTX-A的高灵敏检测方法。所建立的分析方法成功用于实际海水样品中BTX-A含量的分析和监测,结合福建省海洋与渔业局所发布的赤潮监测预警信息,可作为赤潮预警因子有效提高赤潮的监测和预警。

## Abstract (en)

Red tides are a type of natural marine disaster caused by harmful algae characterized by a high toxicity, wide distribution, and long duration. Since the concentration of algal toxins in seawater increases with the occurrence of red tides, algal toxins detected in seawater could be used to predict the occurrence and evolution of red tides. Brevetoxin-A (BTX-A) is a secondary metabolite produced by the harmful algae *Karenia brevis*, whose detection in seawater could form the basis of an accurate warning system for incoming red tides. However, due to the inherent complexity of the seawater matrix and the extremely low levels of BTX-A in seawater, the use of instruments for its direct detection is difficult. Therefore, there is an urgent need to develop a sample pretreatment method for the efficient enrichment of BTX-A in seawater. In this study, a metal-organic backbone material (UiO-66) and its composite with silica microspheres (SiO_2_@UiO-66) were successfully synthesized using the solvothermal method. The prepared SiO_2_@UiO-66 exhibited good hydrophilicity, water stability, and large specific surface area. Furthermore, it also exhibited hydrogen bonding and electrostatic interactions with BTX-A, had a strong affinity for BTX-A, and was able to efficiently adsorb BTX-A in complex matrices. Therefore, SiO_2_@UiO-66 showed potential as a novel packing material for the extraction of BTX-A from solid phase extraction columns. Combined with high performance liquid chromatography-tandem mass spectrometry (HPLC-MS/MS), a highly sensitive detection method for the determination of BTX-A in marine water was established. The established analytical method had a low detection limit (3.0 pg/mL), a wide linear range (10.0 -200.0 pg/mL), and a good linear relationship (*R*=0.9992). Combined with the Fujian Province Red Tide Monitoring and Early Warning Information 2021 issued by the Fujian Provincial Oceanic and Fisheries Bureau, the analytical method established herein was successfully applied to analyze and monitor the content of BTX-A in actual seawater samples. This highlights the proposed system’s potential for use as an early warning factor in the monitoring of red tides, representing a simple and fast pretreatment methodology for the detection of BTX-A in seawater.

赤潮是海洋中某些藻类、原生动物或细菌在一定环境条件下快速增长和积累导致水体变色的一种灾难性的生态异常现象^[1]^。赤潮的发生会产生有毒化合物,导致鱼虾等海洋生物在体内积蓄毒素或死亡,造成沿海渔业巨大经济损失,同时产生的有毒化合物会直接或间接影响人类健康^[2,3]^。短裸甲藻毒素(BTX-A)是由有毒藻短裸甲藻产生的神经性贝毒,BTX-A的含量和赤潮暴发具有很高的相关性^[4]^,因此,完善赤潮预警体系特别是对有毒赤潮的预警尤为重要。其中,高效液相色谱-串联质谱联用技术(HPLC-MS/MS)由于其高灵敏度和选择性^[5⇓-7]^,被认为是定量监测海洋生物BTX-A含量最有力的技术之一。

BTX-A在海水中的含量低且存在基质干扰而难以直接进行检测,实现海水中痕量BTX-A的净化和富集是准确测定其含量的必要环节^[8]^。固相萃取(SPE)具有选择性高、富集能力强、操作简便、样品前处理快速等优点^[9]^,适用于大批量海水样品快速预处理系统。SPE填料是SPE技术至关重要的组成部分,决定了SPE方法的选择性和萃取效率^[10]^。作为SPE填料的吸附剂既要具有优异的萃取性能,如大的比表面积、良好的水稳定性及化学稳定性,还要具有微米级以上的尺寸。

金属有机框架(MOFs)是一系列新型材料,由于其具有开放的金属位点、可调节的孔径和超大的比表面积,而受到高度关注和不断发展^[11⇓-13]^。UiO-66以其大表面积、高热稳定性和水稳定性而闻名,其结构中的ZrO键因具有强大的黏合性,使得该材料能够显著抵抗多种溶剂的侵蚀。由于BTX-A通常以低含量(1~20 ng/L)存在于盐含量高的复杂水体中(海水平均盐度为3.5%),同时HPLC-MS/MS分析BTX-A需要低盐、高纯度或高质量的BTX-A样品,因此需要发展可用于分离富集的高效SPE填料^[14]^。直接使用纳米级锆基UiO-66作为固相萃取吸附剂可行性不高,因其粒径较小,在萃取过程中会形成超高压。因此,制备微米级锆基UiO-66及其复合材料可以解决这一难题。SiO_2_微球经常被用作优选的载体,在SiO_2_微球上生长UiO-66制备锆基复合材料SiO_2_@UiO-66作为萃取吸附剂是克服这些问题的有效策略。

本实验采用溶剂热法制备了高吸附性能、水稳定性强的锆基复合材料SiO_2_@UiO-66,将其填装成SPE柱,并对其结构、性能进行了详细表征。通过对影响SPE萃取效果的各因素进行优化,结合HPLC-MS/MS,建立了用于富集与检测不同时期海水中BTX-A的分析方法。结合福建省海洋与渔业局公布的信息,此方法有望用于预测海域中赤潮的发生。

## 1 实验部分

### 1.1 仪器、试剂与材料

JEOL JSM-6300F扫描电子显微镜(日本JEOL公司); D8 Advance X射线衍射仪(德国Bruker公司); Nicolet iS50傅里叶红外光谱仪、TSQ Quantum Access Max液相色谱-三重四极杆质谱仪(美国Thermo Fisher公司); ASAP2020物理吸附仪(美国Micromeritics公司); Malvern11108 Zeta电位仪(中国Malvern公司); JC2000C接触角测量仪(上海中晨公司); WH10-8602盐度计(大连贝尔分析仪器有限公司); MSW1-3872固相萃取仪(深圳市一正科技有限公司)。

氯化锆(ZrCl_4_,分析纯)、对苯二甲酸(PTA,分析纯)、二氧化硅(SiO_2_, 25 μm,分析纯)、氢氟酸(HF,纯度40%)、乙酸(CH_3_COOH,纯度98%)、3-氨丙基三乙氧基硅烷(APTES,分析纯)、甲酸铵(NH_4_HCO_2_,分析纯)和乙酸铵(CH_3_COONH_4_,分析纯)购自上海阿拉丁试剂有限公司;*N*,*N*-二甲基甲酰胺(DMF,分析纯)和乙醇(EtOH,色谱纯)购自上海麦克林试剂有限公司;甲醇(MeOH,色谱纯)、甲酸(FA,色谱纯)和乙腈(ACN,色谱纯)购自北京百灵威试剂有限公司;实验用水分别为Milli-Q净水器所制得的超纯水(18.2 MΩ·cm)。

BTX-A(纯度≥95%)购自伊普瑞斯科技有限公司。将BTX-A(100.0 mg)溶解在1.0 mL EtOH/H_2_O(4∶1, v/v)中,制成BTX-A的标准储备溶液(100.0 mg/L),然后在-20 ℃下保存。系列标准溶液由EtOH/H_2_O(4∶1, v/v)逐级稀释获得,在4 ℃下保存。

### 1.2 锆基MOFs材料的合成

#### 1.2.1 UiO-66的合成

UiO-66的合成方法在文献[15]的基础上进行优化。将0.6 mmol PTA和0.5 mmol三乙胺溶解在140.0 mL DMF中,搅拌10 min,加入0.36 mol乙酸,在油浴中加热至120 ℃;再将0.6 mmol ZrCl_4_溶解在10.0 mL DMF中,加入至上述溶液中,120 ℃油浴反应6 h。将所得产物用甲醇洗涤离心3次,60 ℃下干燥12 h后,获得UiO-66材料。

#### 1.2.2 SiO_2_@UiO-66的合成

SiO_2_@UiO-66的合成方法示意图如图1所示,为去除SiO_2_封端的烷基,用10.0 mL质量分数为10%的HF刻蚀1.50 g 25 μm SiO_2_1 min。将所得产物用甲醇洗涤离心3次,在60 ℃下干燥12 h后,获得刻蚀后的SiO_2_。在文献[16]的基础上略做修改,将0.50 g 25 μm刻蚀后的SiO_2_溶解在50.0 mL乙醇中,加热搅拌下加入2.0 mL APTES,将所得产物用乙醇洗涤离心3次,干燥,获得SiO_2_-NH_2_;称量0.64 g ZrCl_4_和0.50 g SiO_2_-NH_2_溶解在80.0 mL DMF中,在水浴中保持25 ℃,持续搅拌1 h; 将0.46 g PTA溶解在40.0 mL DMF中,再加入4.0 mL乙酸,搅拌均匀后加入上述溶液中,120 ℃油浴反应24 h,将所得产物用甲醇洗涤,干燥,获得SiO_2_@UiO-66材料。

**图1:**
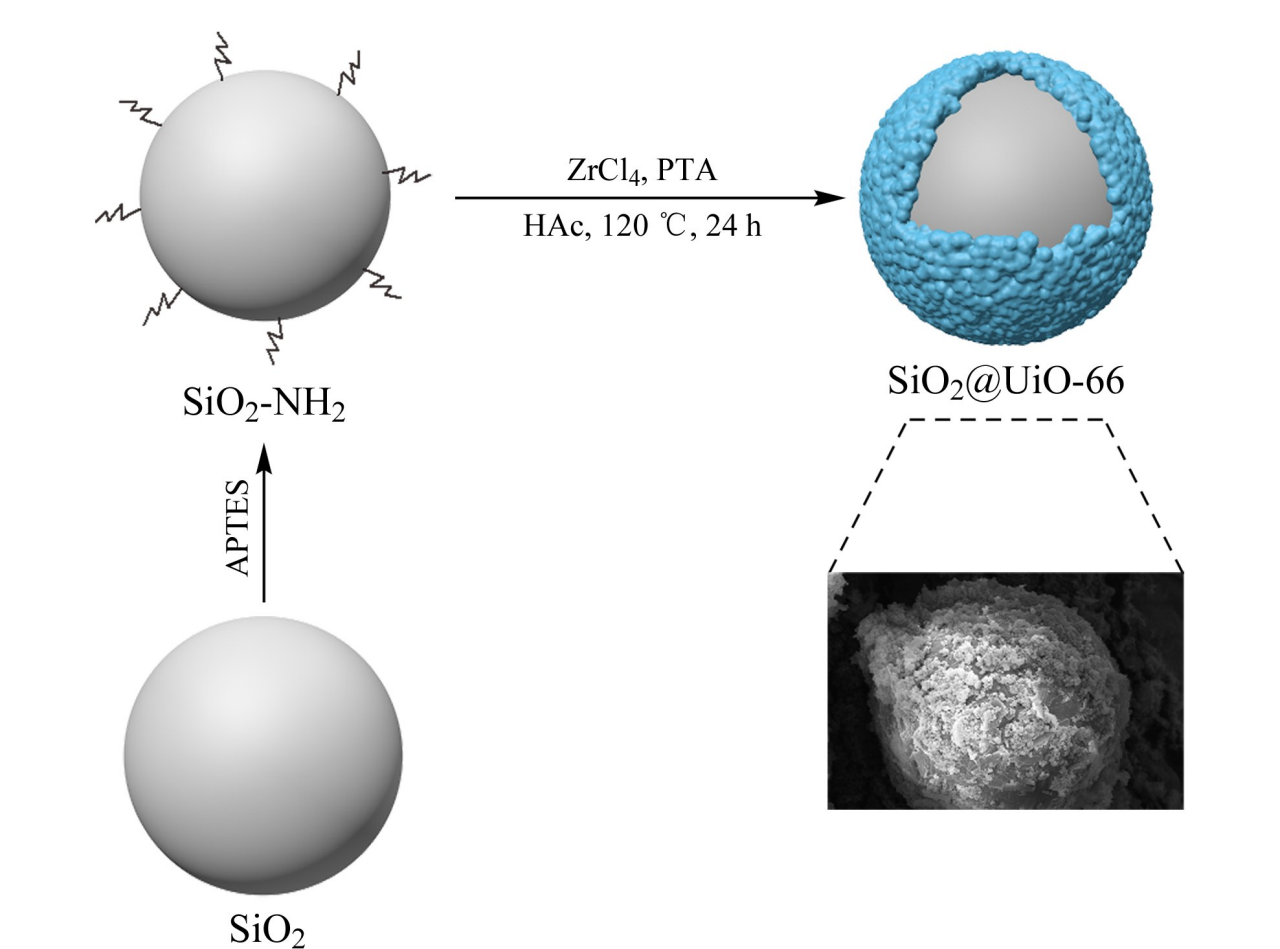
SiO_2_@UiO-66制备过程示意图

### 1.3 样品前处理

于2021年6~9月,采集福建省平潭县东海岸水产养殖业发达的柳水镇柳水码头附近的海水。将取得的海水样品静置1天,沉淀泥沙。用0.22 μm水相滤膜过滤静置后的海水3次,滤膜上没有肉眼可见的杂质即可。将过滤后的海水存放于4 ℃冰柜。

### 1.4 填充柱

用研钵将SiO_2_@UiO-66研磨成均匀粉末后,称量0.20 g。取6 mL SPE柱固定在SPE装置上,先将下筛板装入SPE柱底部,连接水泵,并打开开关。当SPE装置压力达到0.08 MPa时,将称量好的SiO_2_@UiO-66材料装入柱中,通过压力将材料压实平整,最后装入上筛板,压实。

### 1.5 固相萃取

移取300 mL水样,上样至先加入10 mL甲醇活化、后加入10 mL纯水平衡后的SPE柱。上样时压力设为0.08 MPa。上样后用氮气吹干SPE柱,用5.0 mL纯水淋洗,用氮气吹干SPE柱,用2.5 mL含50 mmol/L甲酸铵的甲醇溶液洗脱,收集洗脱液,在35 ℃下氮吹浓缩,用水稀释至0.5 mL,待HPLC-MS/MS测定。

### 1.6 分析条件

色谱柱:Thermo Fisher Hypersil GOLD aQ型色谱柱(150 mm×2.1 mm, 5 μm);流动相:含0.1%甲酸的50 mmol/L甲酸铵水溶液-甲醇(5∶95, v/v),等度洗脱;流速:200 μL/min;柱温:室温;进样量:10 μL。

离子源:电喷雾电离(ESI),正离子模式;喷雾电压:3000 V;汽化室温度:300 ℃;毛细管温度:325 ℃;辅助气:氮气(纯度99.999%), 3000 MPa;鞘气:氮气(纯度99.999%), 1000 MPa;数据采集模式:选择反应监测模式(SRM)。BTX-A的母离子、子离子和SRM碰撞能分别为*m/z* 867.5、*m/z* 849.5和13 eV。用Thermo FisherLC quan 2.7软件采集数据并进行分析。

## 2 结果与讨论

### 2.1 UiO-66和SiO_2_@UiO-66的表征

采用扫描电子显微镜对UiO-66和SiO_2_@UiO-66材料粉末进行形貌表征,由图2a可以看出,合成的UiO-66颗粒具有规则的多面体结构,是UiO-66的八面体典型结构,形貌较好,晶型尖锐,晶体大小较为均一,大小在1 μm左右。由图2b可以看出,每一个SiO_2_上都包覆了UiO-66颗粒,且大部分SiO_2_上包覆的晶体颗粒都较多,能够完整地覆盖其表面。说明SiO_2_@UiO-66制备成功。且从图中可以看出,SiO_2_@UiO-66直径在15~20 μm左右。

**图2:**
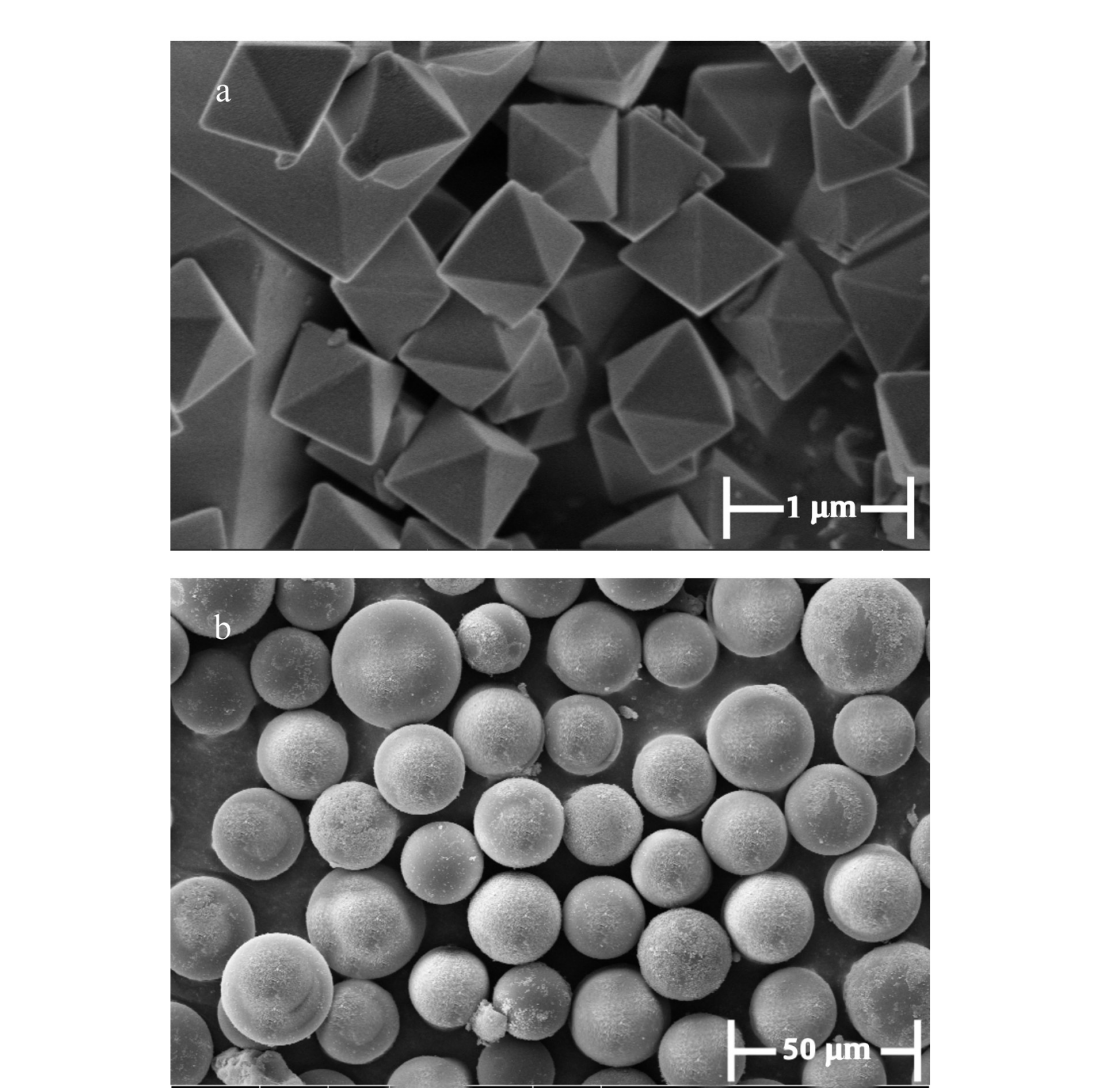
(a)UiO-66和(b)SiO_2_@UiO-66的扫描电子显微镜图

用X射线衍射仪分析UiO-66和SiO_2_@UiO-66的晶体结构(见图3a)。SiO_2_@UiO-66的XRD图谱仍有明显的UiO-66特征峰,且峰形尖锐,同时在10°~20°处有一个很宽的弥散峰为SiO_2_的特征峰。图中峰形与文献[17]的谱图基本一致,该图谱同时拥有UiO-66和SiO_2_的特征峰,说明UiO-66成功生长在SiO_2_上,晶形较好。

**图3:**
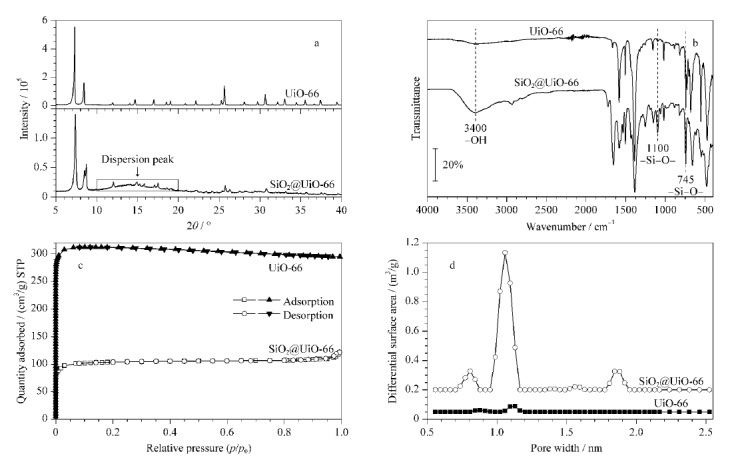
UiO-66和SiO_2_@UiO-66的(a)XRD分析图、(b)红外光谱图、(c)氮气吸附脱附等温线图和(d)孔径分布图

用红外光谱对UiO-66和SiO_2_@UiO-66的基团进行表征(见图3b)。除了UiO-66的特征吸收峰外,在3400 cm^-1^处出现了较宽的吸收峰,对应于-OH的反对称伸缩振动和对称伸缩振动,1100 cm^-1^和745 cm^-1^处出现的强吸收谱峰属于-Si-O-的特征吸收峰,说明合成的产物为SiO_2_@UiO-66^[18]^。

通过氮气吸附-脱附仪对UiO-66和SiO_2_@UiO-66的比表面积(见图3c)和孔径大小(见图3d)进行测定。UiO-66和SiO_2_@UiO-66的吸脱附等温线均为典型的Ⅰ型等温线^[19]^, UiO-66的比表面积为899.1 m^2^/g, SiO_2_@UiO-66的比表面积为310.9 m^2^/g。UiO-66是一种金属有机骨架材料,具有超大的比表面积、良好的孔结构和可调控性^[12]^。相比之下,SiO_2_@UiO-66形成的复合物的比表面积可能会减小,这是因为硅球与UiO-66之间的键合作用,使得原本UiO-66中可供外部接触的有效表面积在一定程度上被覆盖或减小,从而减少了复合物SiO_2_@UiO-66的总有效接触面积。因此,UiO-66的这些特性使UiO-66具有更大的比表面积和更高的BTX-A最大吸附量(*Q*_max_)。UiO-66材料孔径尺寸主要为1.17 nm和0.87 nm, SiO_2_@UiO-66材料孔径尺寸大小主要分布在1.05 nm左右,在0.81、1.56、1.81 nm处也有少量分布,孔径分布范围较大。其等温线型和孔径大小都表明UiO-66和SiO_2_@UiO-66为微孔材料。

用接触角测量仪对UiO-66和SiO_2_@UiO-66进行亲疏水性测试(见图4a)。可以看到,UiO-66水接触角为58.7°, SiO_2_@UiO-66水接触角为55.6°,水接触角在90°以下均为亲水材料^[20]^,因此UiO-66和SiO_2_@UiO-66均具有亲水性,在水中具有良好的分散性和稳定性,为吸附海水中的BTX-A提供了优越的前提条件。

**图4:**
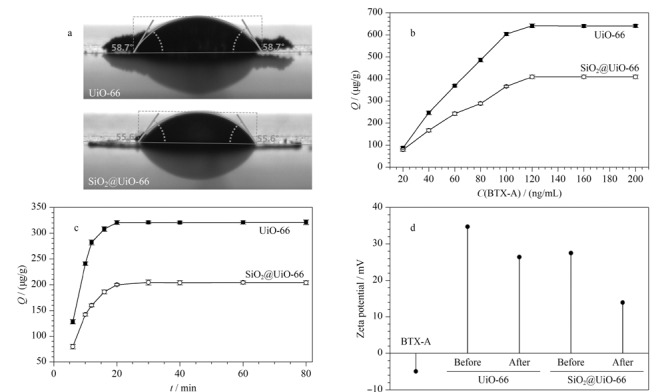
UiO-66和SiO_2_@UiO-66的(a)水接触角、(b)吸附等温线(*n*=3)、(c)吸附动力学曲线(*n*=3)和(d)吸附前后的Zeta电位

通过吸附动力学和热力学实验研究UiO-66和SiO_2_@UiO-66材料的吸附行为(见图4b和4c)。可以看到,吸附等温线图中UiO-66对BTX-A的*Q*_max_为641.0 μg/g, SiO_2_@UiO-66对BTX-A的*Q*_max_为409.8 μg/g。两种材料均对BTX-A有较高的吸附容量和优异的吸附性能,可能原因是:(1)UiO-66和SiO_2_@UiO-66与BTX-A之间存在较强的静电作用和氢键作用;(2)UiO-66和SiO_2_@UiO-66较大的比表面积与BTX-A大小相近的孔径;(3)两种材料都具有较好的亲水性,在水中的分散性较好,可增加材料与BTX-A的接触面积;(4)UiO-66主结构与BTX-A的苯环形成强烈的*π*-*π*和疏水相互作用;(5)在吸附动力学实验中,UiO-66和SiO_2_@UiO-66都在20 min时达到吸附平衡,说明两种材料对BTX-A的吸附速度较快,有较好的吸附性能。

通过Zeta电位仪测试材料吸附BTX-A前后及BTX-A的电位值探究UiO-66和SiO_2_@UiO-66与BTX-A之间是否有静电作用(见图4d)。由于SiO_2_的Zeta电位值为负,SiO_2_@UiO-66的电位值比UiO-66电位值有所降低。很明显可以看到与目标物结合后的电位值降低,说明UiO-66和SiO_2_@UiO-66与BTX-A结合为静电作用。

### 2.2 SPE条件优化

为了使SPE对BTX-A的萃取达到最优,本工作对影响萃取的诸多因素进行了考察,包括上样量、淋洗剂体积、洗脱剂、洗脱剂体积和柱吸附容量等。

#### 2.2.1 上样量

为了提高样品前处理的速度,缩短富集BTX-A的时间,需要优化SPE柱的上样量。实验以纯水为上样溶剂,配制质量浓度为50.0 pg/mL的BTX-A标准溶液,其溶液量分别为100.0、200.0、300.0、400.0、500.0 mL。先用10.0 mL甲醇活化,再用10.0 mL水平衡UiO-66与SiO_2_@UiO-66 SPE柱,再将上述溶液作为上样样品用SPE柱萃取,记录上样时间,检测萃取后BTX-A的回收率,结果见图5a。当上样体积为300 mL时,SiO_2_@UiO-66 SPE柱的上样时间为30 min,回收率达到最大值。UiO-66 SPE柱则因为材料粒径太小,柱压过高,上样时间均超过30 min。

**图5:**
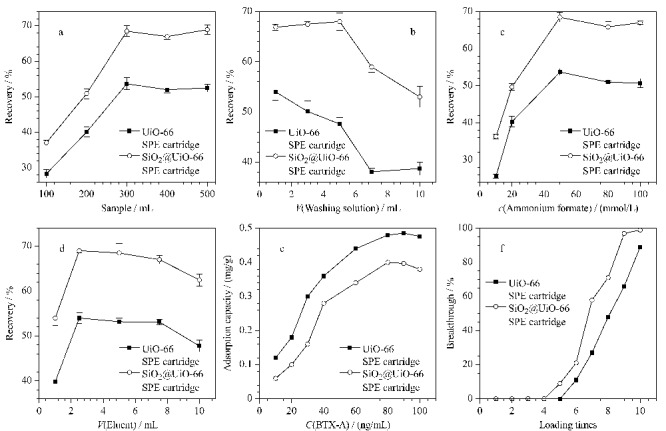
(a)上样量、(b)淋洗剂体积、(c)洗脱液中甲酸铵浓度和(d)洗脱剂体积的优化,以及UiO-66和 SiO_2_@UiO-66SPE柱(e)最大吸附量和(f)渗漏实验(*n*=6)

#### 2.2.2 淋洗剂体积

淋洗剂的体积也是SPE中的关键,淋洗剂用量过多会造成目标物被淋洗,但是用量过少会造成淋洗不彻底,对后续用HPLC-MS/MS检测的结果造成影响^[21]^。实验考察了淋洗液体积为1.0、3.0、5.0、7.0、10.0 mL时的效果,结果见图5b。对UiO-66 SPE柱,淋洗液体积超过1.0 mL时,淋洗液就会洗脱一部分目标物,后续检测的回收率偏低。对SiO_2_@UiO-66 SPE柱,淋洗液量超过5.0 mL时,淋洗液就会洗脱一部分目标物,后续检测的回收率偏低。因此,实验以5.0 mL为最佳淋洗剂体积。

#### 2.2.3 洗脱剂

洗脱剂是与目标物结合作用更强的物质,通过破坏SPE柱中填料与BTX-A的作用力来洗脱目标物^[22]^。选择极性较强的甲醇溶剂,能够破坏静电作用,在其中加入弱酸性的甲酸铵也能加强其破坏效果^[23]^。同时,BTX-A对甲醇有较强的亲和力,极易溶于甲醇中^[24]^。因此,实验在甲醇溶剂中加入不同浓度(10、20、50、80、100 mmol/L)的甲酸铵作为洗脱剂,结果见图5c。以含50 mmol/L甲酸铵的甲醇溶液的洗脱效果最好,通过UiO-66柱与SiO_2_@UiO-66 SPE柱的样品洗脱后回收率分别在50%和60%以上。

#### 2.2.4 洗脱剂体积

实验其他条件保持不变,选择洗脱剂体积分别为1.0、2.5、5.0、7.5、10.0 mL。实验证明,当洗脱剂体积为1.0 mL时,回收率低于30%,说明没有完全洗脱。当洗脱剂为2.5和5.0 mL时,经SiO_2_@UiO-66 SPE柱的样品回收率均达到60%以上,其中用2.5 mL洗脱剂洗脱的样品检测回收率高达66.0%,其回收率较为理想。当洗脱剂大于5.0 mL后,回收率下降。如图5d所示,实验以2.5 mL含50 mmol/L甲酸铵的甲醇溶液为最佳洗脱条件。

#### 2.2.5 柱吸附容量

由于UiO-66和SiO_2_@UiO-66都可以对BTX-A产生吸附,且吸附效果较好,本实验采用将材料填入SPE柱中萃取BTX-A的方法,考察SPE柱的吸附容量。配制10.0~100.0 ng/mL的BTX-A标准溶液,研究UiO-66与SiO_2_@UiO-66 SPE柱对BTX-A的吸附量(见图5e)。随着BTX-A质量浓度的增加,UiO-66与SiO_2_@UiO-66 SPE柱对BTX-A的吸附量也一同增加。当质量浓度为80.0 ng/mL时,SiO_2_@UiO-66柱的吸附量达到平衡,而UiO-66柱在90 ng/mL时吸附量达到平衡,这是由于SiO_2_@UiO-66的比表面积小于UiO-66的比表面积。

#### 2.2.6 渗漏实验

渗漏实验是通过连续上样,收集过滤液检测是否含有目标物的实验,是对SPE柱最大样品承载能力的测试。用纯水作为上样溶剂,配制1.0 μg/L的BTX-A标准溶液。每次上样量为10.0 mL,用HPLC-MS/MS检测每一次上样后被SPE柱萃取的上清液(见图5f)。UiO-66 SPE柱在上样10次后开始渗漏,SiO_2_@UiO-66 SPE柱在上样8次后开始渗漏。这两种SPE柱对BTX-A都有较大的吸附容量。

### 2.3 方法学考察

以处理过后的海水样品为溶剂,配制5.0~200.0 pg/mL的BTX-A标准品溶液,以空白海水样品为对照,用SiO_2_@UiO-66 SPE柱在最优条件下进行SPE-HPLC-MS/MS分析,得到海水中BTX-A分析方法的检出限(信噪比≥3时的最低加标水平)为3.0 pg/mL,线性范围为10.0~200.0 pg/mL,线性回归方程为*y*=0.03239*x*-0.0148(*R*=0.9992)。

### 2.4 实际样品分析

为进一步评价所建立方法的稳定性和可行性,将所建立的方法用于海水样品的分析,结果均未检测到BTX-A(见表1)。对实际海水样品进行加标回收试验(分别加入10、50、100 pg/mL的BTX-A),每个样品平行检测3次。结果见表1,BTX-A的平均回收率为80.3%~108.7%, RSD≤8.5%。由实际样品加标后的色谱图(图6)可知,海水虽然基质复杂,但总体不干扰测定,且在较低浓度下也有较好的抗干扰能力。 因此,所建立的分析方法可用于海水中BTX-A的检测。

**表1:** 实际样品检测结果(*n*=3)

Sample No.	Date	Found/ (pg/mL)	Recoveries/%			RSD^*^/%
			10 pg/mL	50 pg/mL	100 pg/mL	
1	6.70	N. D.	87.2	98.3	82.5	7.7
2	6.15	N. D.	94.4	92.5	86.5	7.3
3	6.21	N. D.	88.1	92.7	103.7	7.3
4	6.28	N. D.	94.2	93.4	103.9	6.7
5	7.19	N. D.	80.3	107.5	91.2	8.1
6	8.16	N. D.	87.9	81.7	96.3	8.5
7	9.17	N. D.	108.7	108.5	99.8	7.4

N. D.: not detected. * RSD of recovery at 50 pg/mL.

**图6:**
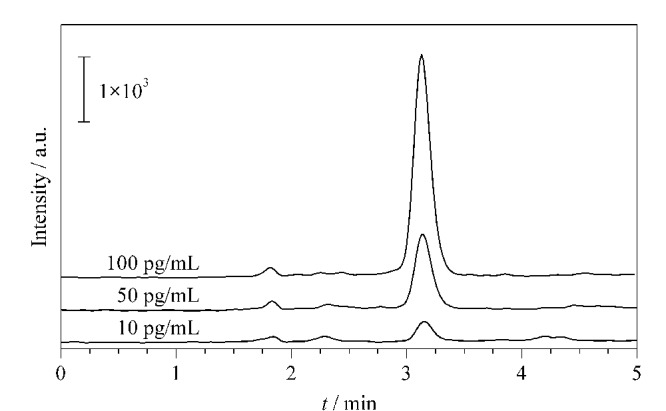
实际海水样品的加标色谱图

## 3 结论

本工作通过溶剂热法成功制备了SiO_2_@UiO-66复合材料,该材料以包覆硅球为载体,结合Zr-MOFs的结构优势,展现出高的比表面积、优异的亲水特性以及强大的吸附能力。将此复合材料作为SPE柱的填料,建立了SPE-HPLC-MS/MS分析BTX-A的方法。 SiO_2_@UiO-66 SPE柱萃取海水中的BTX-A是一种极为高效的分离富集方法,为海洋水体复杂基质中赤潮毒素的检测提供了一种方便快捷的样品前处理技术,有一定的可行性和较强的实用性。
